# Downregulation of vasohibin-2, a novel angiogenesis regulator, suppresses tumor growth by inhibiting angiogenesis in endometrial cancer cells

**DOI:** 10.3892/ol.2013.1119

**Published:** 2013-01-08

**Authors:** TAKAHIRO KOYANAGI, YASUSHI SAGA, YOSHIFUMI TAKAHASHI, YASUHIRO SUZUKI, MITSUAKI SUZUKI, YASUFUMI SATO

**Affiliations:** 1 Department of Obstetrics and Gynecology, School of Medicine, Jichi Medical University, Shimotsuke-shi, Tochigi 329-0498;; 2 Department of Vascular Biology, Institute of Development, Aging and Cancer, Tohoku University, Aoba-ward, Sendai 980-8575, Japan

**Keywords:** endometrial cancer, vasohibin-2, tumor angiogenesis, molecular-targeted therapy, endothelial cells

## Abstract

The vasohibin-2 (VASH2) gene was originally found to be expressed in infiltrating mononuclear cells of a mouse model of hypoxia-induced subcutaneous angiogenesis. These cells are mobilized from bone marrow to promote angiogenesis. Recently, VASH2 has been demonstrated to be expressed in several types of cancer in which it promotes tumor development through angiogenesis. However, its role in endometrial cancer remains unknown. Using quantitative reverse transcription-polymerase chain reaction (RT-PCR), we found that VASH2 was overexpressed in several human endometrial cancer cell lines, including the HEC50B cell line, which we used to further examine the role of VASH2. Although knockdown of VASH2 with stable transfection of shRNA had little effect on the proliferation of HEC50B cells *in vitro*, knockdown in an *in vivo* murine xenograft model inhibited tumor growth by decreasing tumor angiogenesis. In addition, the supernatant from HEC50B cells that expressed VASH2 significantly promoted the proliferation of human umbilical vein endothelial cells. By contrast, knockdown of VASH2 significantly attenuated the proliferative effect. These results indicate that VASH2 contributes to the development of endometrial cancer by promoting angiogenesis through a paracrine mode of action. Consequently, VASH2 may be considered to be a novel molecular target for endometrial cancer therapy.

## Introduction

Endometrial cancer is the most frequent gynecological malignancy and the fourth most common type of cancer in females in the United States (1). When endometrial cancer is localized to the uterus, it is often detected at an early stage; therefore, the overall survival rate is >80% (1). However, the prognosis of advanced endometrial cancer remains poor (2). Surgery, radiotherapy and multidrug chemotherapy have all been used to treat advanced endometrial cancer with little success. Therefore, limited improvements in overall treatment outcomes have been observed in endometrial cancer over the past 30 years (1). Therefore, novel strategies, such as anti-angiogenic therapy and targeted molecular therapy, may be useful in improving the prognosis of endometrial cancer.

Angiogenesis is a hallmark of malignant tumor development, and is important in the growth of primary, metastatic and disseminated lesions of endometrial cancer (3). Therefore, anti-angiogenic therapy may be effective in treating endometrial cancer. At present, anti-angiogenic therapy has been approved for several types of cancer, including colon, lung, breast and kidney cancer. Furthermore, several drugs that target vascular endothelial growth factor (VEGF) signals are already in clinical use (4). While the effectiveness of these drugs is encouraging, resistance to anti-angiogenic therapy has been demonstrated in several reviews (5–8). To overcome these problems, novel molecular targets for anti-angiogenic therapy need to be discovered.

The vasohibin family includes vasohibin-1 (VASH1) and vasohibin-2 (VASH2). In endothelial cells (ECs), VASH1 is selectively induced by angiogenesis stimulators, such as VEGF and basic fibroblast growth factor (bFGF). VASH1 functions as an intrinsic negative feedback regulator at the termination zone of angiogenesis (9). VASH2 is a homolog of VASH1, and the VASH2 gene is highly expressed in bone marrow-derived mononuclear cells and weakly expressed in ECs. In contrast to VASH1, VASH2 has been found to promote angiogenesis at the sprouting front in a mouse model of hypoxia-induced subcutaneous angiogenesis (10). To date, there are a limited number of studies available in the literature concerning the correlation between VASH2 and tumor angiogenesis (11,12). Recently, we demonstrated that VASH2 contributes to the development of tumor growth and peritoneal dissemination by promoting angiogenesis in human ovarian serous adenocarcinoma (12). However, the specific role of VASH2 in endometrial cancer remains unknown.

In this study, we used a short hairpin RNA (shRNA) vector to silence VASH2 expression in a VASH2-expressing endometrial cancer cell line, to further elucidate the relationship between VASH2 expression and endometrial cancer progression. Moreover, we investigated the function of VASH2 in endometrial cancer angiogenesis to develop a VASH2-targeted anti-angiogenic molecular therapy for endometrial cancer.

## Materials and methods

### Cell culture

Human endometrial cancer cell lines, HEC1A and HEC50B, were obtained from the Japanese Collection of Research Bioresources (JCRB; Osaka, Japan), and were maintained as described previously (13,14). The Ishikawa cell line (clone 3H12) was a gift from Dr M. Nishida (Department of Obstetrics and Gynecology, National Hospital Organization, Kasumigaura Medical Center, Ibaraki, Japan), and was maintained as described previously (15). Cells were cultured in RPMI-1640 medium (Wako Pure Chemical Industries, Ltd., Osaka, Japan) supplemented with 10% heat-inactivated fetal bovine serum (FBS; BioWest S.A.S, Nuaillé, France). Human umbilical vein endothelial cells (HUVECs) were obtained from Kurabo Industries, Ltd. (Osaka, Japan) and were cultured in type I collagen-coated dishes (Iwaki, Chiba, Japan) in endothelial basal medium (EBM)-2 (Lonza, Walkersville, MD, USA) supplemented with EGM-2-MV-SingleQuots (Lonza) containing VEGF, bFGF, insulin-like growth factor-1, epidermal growth factor and 5% FBS. All cells were cultured at 37°C in a humidified atmosphere with 5% CO_2_.

### shRNA stable cell line and control cell line

The DNA oligo-nucleotide sequences encoding shRNA targeting VASH2 included forward: 5′-CACGGGGCAGATTATAAGAATTACGTGTGCTGTCCGTAATTCTTGTAGTCTGCTCCTTTTT-3′ and reverse: 5′-CCCCGTCTAATATTCTTAATGCACACGACAGGCATAAGAACATCAGACGAGGAAAAATACG-3′. The oligonucleotides were synthesized, annealed and inserted into the *Bsp*MI site of the piGENE PURhU6 vector (16), which contained a human U6 promoter and a puromycin resistance gene. The shRNA expression plasmid (piGENE PURhU6/shVASH2) and control plasmid (piGENE PURhU6) were transfected into HEC50B cells by the use of Lipofectamine LTX and Plus Reagent (Invitrogen Life Technologies, Carlsbad, CA, USA) according to the manufacturer’s instructions. Following transfection, the cells were selected in puromycin-containing medium (Calbiochem, La Jolla, CA, USA). Subsequently, VASH2-knockdown clones and control clones were established.

### Reverse transcription-polymerase chain reaction (RT-PCR)

Total RNA was extracted from cell cultures using Isogen (Nippon Gene, Toyama, Japan) according to the manufacturer’s instructions. The concentration of extracted RNA was determined using the Nanodrop 2000c spectrophotometer (Thermo Scientific, Wilmington, DE, USA). First-strand cDNA was generated with ReverTra Ace (Toyobo Co., Ltd., Osaka, Japan). The RT-PCR procedure was performed in a DNA thermal cycler (Takara Bio, Inc., Tokyo, Japan). PCR conditions consisted of an initial denaturation step at 94°C for 5 min, followed by 35 cycles comprising a 15-sec phase at 94°C (denaturation), a 30-sec phase at 56°C (annealing) and a 30-sec phase at 72°C (extension). PCR products were separated on a 2% agarose gel and visualized under ultraviolet rays by ethidium bromide staining. The primer pairs used were as follows: forward, 5′-ACCACAGTCCATGCCATCAC-3′ and reverse, 5′-TCCACCACCCTGTTGCTGTA-3′ for human GAPDH gene; and forward, 5′-ACGTCTCAAAGATGCTGAGG-3′ and reverse, 5′-CTCTCCGACCCAAGTGAGAA-3′ for human VASH2.

### Quantitative real-time RT-PCR

cDNA was synthesized as described previously. The specific primer for human VASH2 was the same as that for RT-PCR. The CFX96 real-time PCR detection system was used with the reagent SYBR Premix Ex Taq™ (Takara Bio, Inc.) for real-time PCR. PCR conditions included an initial denaturation step at 95°C for 3 min, followed by 50 cycles comprising a 10-sec phase at 95°C, a 10-sec phase at 56°C and a 30-sec phase at 72°C. Amplification of GAPDH was used as an endogenous control. Relative gene expression levels were calculated using the comparative Ct method.

### Proliferation of tumor cells

Proliferation of tumor cells was measured by performing the TetraColor ONE cell proliferation assay (17). Briefly, cells were seeded at a density of 2×10^3^ cells/well in a 96-well plate and incubated at 37°C. After 24, 48, 72 and 96 h, 5 *μ*l of TetraColor ONE (Seikagaku Co., Tokyo, Japan) was added to each well. The mixture was subsequently incubated for an additional 2 h and absorbance at 450 nm was monitored.

### Proliferation of ECs

The VASH2-knockdown clones of HEC50B and HEC50B cells transfected with empty vector were cultured for 24 h. The conditioned medium (CM) was obtained from each culture. Subsequently, cellular components were removed from the CM using a Millex GP filter (0.22 *μ*m; PES, 33MM; Millipore, Billerica, MA, USA). HUVECs were plated in a 96-well plate at a density of 2×10^3^ cells/well and cultured in medium containing the CM obtained previously. After 48 h, the proliferation of HUVECs was measured using the TetraColor ONE as previously described.

### Mouse xenograft model of human endometrial cancer

Female 6- to 8-week old BALB/c nude mice were obtained from CLEA Japan, Inc. (Tokyo, Japan). The experimental protocols were approved and conducted according to the guidelines for animal experimentation of Jichi Medical University. Tumor cells were subcutaneously transplanted into the back of mice at a concentration of 4×10^6^ cells/mouse. The dimensions of the tumor were measured twice per week and the volume was calculated using the following formula: Volume=1/2× (long diameter)x(short diameter)^2^. For the immunohistochemical analysis of tumor angiogenesis, the tumors were frozen in optimal cutting temperature (OCT) compound (Sakura, Tokyo, Japan), cut into 7-*μ*m sections, fixed in methanol for 20 min at −20°C and blocked with 1% bovine serum albumin in phosphate-buffered saline (PBS) for 45 min at room temperature. Primary antibody reactions were performed overnight at 4°C with rat monoclonal antibody against mouse CD31 (BD Biosciences, San Diego, CA, USA) at a dilution of 1:500. Secondary antibody reactions were performed for 1 h at room temperature with Alexa Fluor 488-conjugated donkey anti-rat IgG (Molecular Probes, Eugene, OR, USA) at a dilution of 1:500. After washing 3 times with PBS, the sections were covered with fluorescent mounting medium (Dako, Carpintera, CA, USA). All samples were analyzed with a BZ-9000 fluorescence microscope (Keyence, Osaka, Japan) at room temperature. To evaluate tumor angiogenesis, the vascular luminal area was calculated using five different fields of each tumor section with BZ-H1C software (Keyence).

### Statistical analysis

A Student’s t-test was used to test for a significant difference between the 2 groups. P<0.05 was considered to indicate a statistically significant difference.

## Results

### VASH2 expression

**Figure 1:**
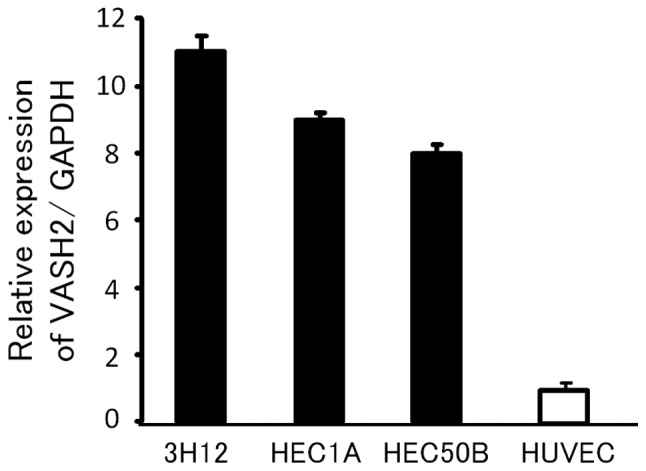
Expression of vasohibin-2 (VASH2) in human endometrial cancer cell lines. Quantitative reverse transcription-polymerase chain reaction (RT-PCR) shows the expression of VASH2 in various cell lines of endometrial cancer. VASH2 expression in human umbilical vein endothelial cells (HUVECs) is defined as 1. Means and standard deviations are shown (n=3).

To examine the possible involvement of VASH2 in endometrial cancer, we analyzed human endometrial cancer cell lines by quantitative RT-PCR. As demonstrated in Fig. 1, VASH2 mRNA expression was considerably higher in several human endometrial cancer cell lines than in the HUVECs.

### Knockdown of VASH2 and its effects in vitro and in vivo

**Figure 2:**
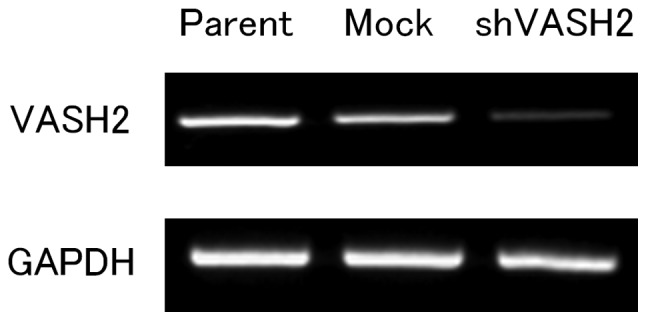
Establishment of vasohibin-2 (VASH2) knockdown clone in the HEC50B cell line. VASH2 knockdown (shVASH2) clones from HEC50B cells and their control mock transfectant were established. Reverse transcription-polymerase chain reaction (RT-PCR) shows the knockdown of VASH2 in shVASH2 clones established from HEC50B cells.

**Figure 3:**
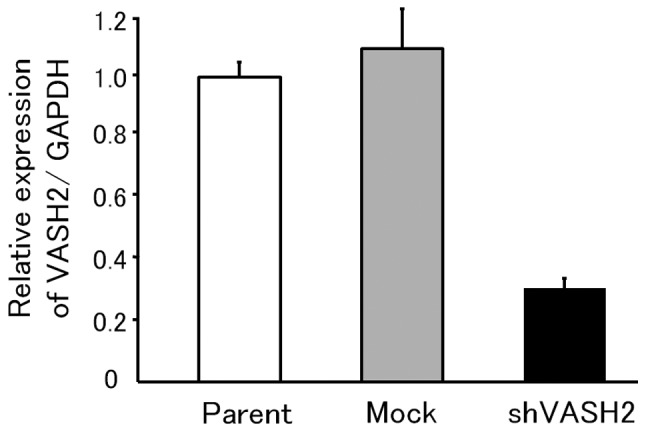
Gene expression of vasohibin-2 (VASH2) in HEC50B transfectants. Quantitative reverse transcription-polymerase chain reaction (RT-PCR) shows the knockdown of VASH2 in VASH2-knockdown (shVASH2) clones with a knockdown efficacy >70%. VASH2 expression in the parent cell line is defined as 1. Means and standard deviations are shown (n=5).

**Figure 4:**
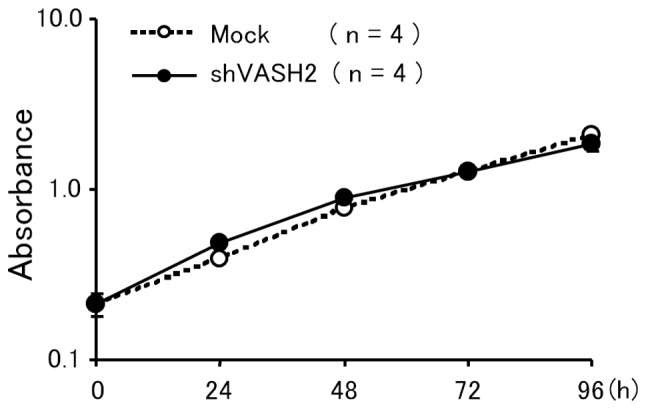
*In vitro* proliferation of HEC50B transfectants. The proliferation of vasohibin-2-knockdown (shVASH2) clones and of their control mock transfectants were compared under the same cell culture conditions *in vitro*. Knockdown of VASH2 did not affect the *in vitro* proliferation of HEC50B. Means and standard deviations are shown (n=4).

**Figure 5:**
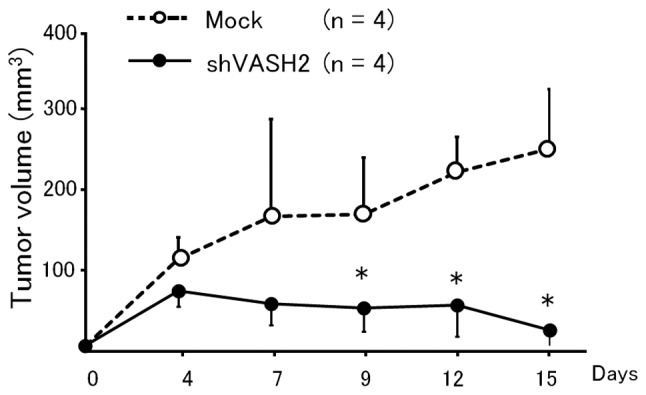
Subcutaneous xenograft model of HEC50B transfectants. Mock or vasohibin-2-knockdown (shVASH2) clones established from HEC50B cells were inoculated subcutaneously into nude mice, and the serial tumor growth was compared in terms of tumor volume. Knockdown of VASH2 significantly inhibited the subcutaneous tumor growth *in vivo*. Means and standard deviations are shown (n=4). ^*^P<0.05 vs. mock transfectants.

**Figure 6:**
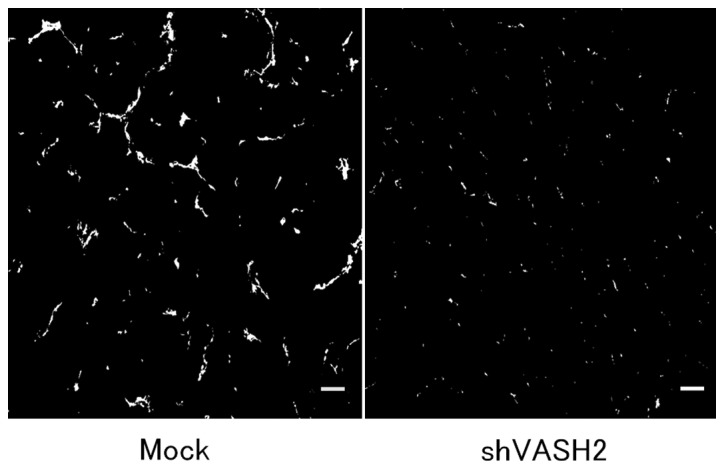
Immunofluorescent staining of CD31 to show tumor angiogenesis. Frozen sections of tumors obtained from mock or HEC50B shVASH2 clones were immunostained with anti-CD31 antibody. Scale bar, 50 *μ*m.

**Figure 7:**
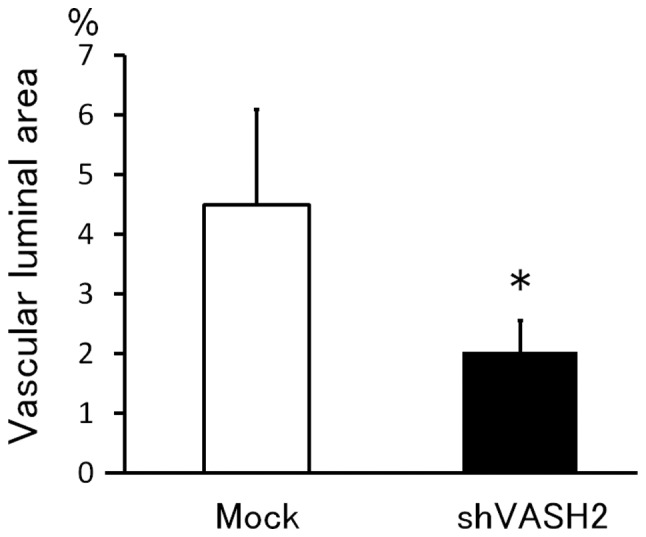
Quantification of tumor angiogenesis. The vascular luminal area was calculated from five different fields of each tumor section and then compared. Tumor angiogenesis decreased significantly in tumors derived from vasohibin-2-knockdown (shVASH2) clones. Means and standard deviations are shown (n=4). ^*^P<0.05 vs. mock.

To clarify the function of VASH2 in endometrial cancer, we performed a loss-of-function experiment by knocking down VASH2 expression. We used HEC50B, an endometrial cancer cell line with high VASH2 expression, for the following experiments. By the transfection of shRNA, we established the VASH2-knockdown (shVASH2) clone from HEC50B (Fig. 2). The efficacy of knockdown was >70% (Fig. 3). Knockdown of VASH2 did not affect the *in vitro* proliferation of HEC50B cells (Fig. 4). We then inoculated the shVASH2 clone subcutaneously into nude mice, and observed a significant inhibition of tumor growth in the shVASH2 group compared with the control mock group (Fig. 5). Furthermore, we analyzed angiogenesis in the tumors of the mouse xenograft model. As expected, tumor angiogenesis was significantly inhibited in the shVASH2 tumors, as assessed by immunofluorescent staining of CD31 (Figs. 6 and 7).

### Effect of secreted VASH2 on EC proliferation

**Figure 8:**
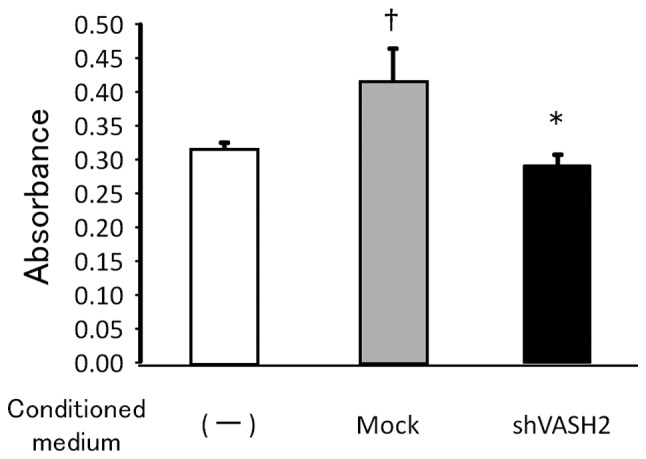
Effect of secreted vasohibin-2 (VASH2) on endothelial cell (EC) proliferation. The proliferation of human umbilical vein endothelial cells (HUVECs) incubated without conditioned medium (CM) or with CM from VASH2-knockdown (shVASH2) clones or mock transfectants of HEC50B cells was compared *in vitro.* Secreted VASH2 promoted the proliferation of HUVECs and knockdown of VASH2 attenuated that effect. Means and standard deviations are shown (n=4). ^†^P<0.05 vs. without CM. ^*^P<0.05 vs. with CM from mock transfectants.

Members of the vasohibin family are secretory proteins that bind to the intracellular small vasohibin-binding protein (SVBP) (18). To investigate the effect of VASH2 on the ECs, we evaluated the proliferation of HUVECs by using the CM from shVASH2 clones or from mock transfectants of HEC50B. As demonstrated in Fig. 8, secreted VASH2 significantly promoted the proliferation of HUVECs, whereas knockdown of VASH2 significantly attenuated the proliferative effect.

## Discussion

In the present study, we examined the correlation between VASH2 and endometrial cancer cell for the first time. VASH2 was expressed in human endometrial cancer cell lines, and the specific knockdown of VASH2 from the endometrial cancer cell line, HEC50B, significantly inhibited tumor growth by decreasing tumor angiogenesis. In addition, the experiment using the CM revealed that secreted VASH2 significantly promoted the proliferation of HUVECs. These results suggest that VASH2 secreted from the cancer cells acts on neighboring ECs to stimulate angiogenesis in a paracrine manner, and thus contributes to the development of endometrial cancer.

Angiogenesis is recognized as a principal hallmark of various types of cancer (19). There are a number of angiogenic stimulators, one of the most important of which is VEGF, which stimulates EC migration and proliferation, as well as EC tube formation. VEGF is the prototype of the VEGF family, and its pro-angiogenic signals are mainly transmitted via its type 2 receptor (VEGFR2) on ECs (20). In endometrial cancer tissues, VEGF expression is associated with elevated tumor vascularization as measured by microvessel density (3), and is a predictive marker for decreased 5-year survival in patients with advanced endometrial carcinoma (21–23). Thus, anti-angiogenic therapy is considered to be a promising option for treating endometrial cancer. Current VEGF-targeted therapeutic drugs, including bevacizumab (a monoclonal antibody against VEGF-A), have yielded promising results in animal models and clinical trials of endometrial cancer (3,24). However, resistance to such therapeutics may occur, owing to the development of compensatory mechanisms for producing angiogenic factors other than VEGF, or the recruitment of bone marrow-derived angiogenic cells. Therefore, alternative targets for anti-angiogenic therapy, a number of which are being investigated, ought to be sought (25). Taking its stimulatory effect on angiogenesis into account, VASH2 may be a novel molecular target for the treatment of endometrial cancer.

While the putative VASH2 receptor and its downstream signaling are currently under investigation, a number of studies have examined the novel function of VASH2. In one study, an autocrine and paracrine mode of action for VASH2 was found to enhance the expression of FGF-2 and VEGF by nuclear factor-κB upregulation in hepatocellular carcinoma (HCC) cells (11). Furthermore, VASH2 has been found not only to accelerate angiogenesis but also to promote HCC cell proliferation (11). In ovarian serous adenocarcinoma cells, the expression of VASH2 was inversely correlated with that of miR-200b, which represses the expression of ZEB1 and ZEB2, the products of which are key to epithelial-to-mesenchymal transition (26). Therefore, VASH2 may possess other tumor-promoting functions, such as invasion and migration. These features of VASH2 require further investigation.

The local balance between angiogenesis stimulators and inhibitors, both of which are activated simultaneously during angiogenesis, determines the occurrence and progression of angiogenesis. In contrast to VEGF and VASH2, VASH1 has been shown to both inhibit EC angiogenesis and protect EC from apoptosis (11). As a VEGF-independent and EC-extrinsic angiogenesis regulator, VASH2 is considered to be a novel target for anti-angiogenic therapy that enables the toxic side effects of anti-VEGF therapy, such as hypertension and proteinuria, to be avoided. Moreover, the twin combination of VASH2 inhibition and VASH1 upregulation would be a powerful anti-cancer strategy.

In summary, VASH2 contributes to the development of endometrial cancer by regulating angiogenesis through paracrine effects. As such, it constitutes a promising molecular target for endometrial cancer therapy.
